# Postoperative changes in ventricular cerebrospinal fluid biomarkers with correlation to clinical outcome in idiopathic normal pressure hydrocephalus

**DOI:** 10.1177/13872877251350308

**Published:** 2025-06-20

**Authors:** Rebecca Grønning, Anna Jeppsson, Per Hellström, Kerstin Andrén, Katarina Laurell, Dan Farahmand, Henrik Zetterberg, Kaj Blennow, Carsten Wikkelsø, Mats Tullberg

**Affiliations:** 1 Hydrocephalus Research Unit, Department of Clinical Neuroscience, Institute of Neuroscience and Physiology, Sahlgrenska Academy, University of Gothenburg, Gothenburg, Sweden; 2 Angered Hospital, SV Hospital Group, Angered, Gothenburg, Sweden; 3 Department of Medical Sciences, Neurology, Linköping University, Linköping, Sweden; 4 Department of Neurology, Sahlgrenska University Hospital, Gothenburg, Sweden; 5 Clinical Neurochemistry Laboratory, Sahlgrenska University Hospital, Mölndal, Sweden; 6 Department of Psychiatry and Neurochemistry, Institute of Neuroscience and Physiology, Sahlgrenska Academy, University of Gothenburg, Gothenburg, Sweden; 7 Department of Neurodegenerative Disease, UCL Institute of Neurology, Queen Square, London, UK; 8 UK Dementia Research Institute at UCL, London, UK; 9 Hong Kong Center for Neurodegenerative Diseases, Clear Water Bay, Hong Kong, China; 10 Wisconsin Alzheimer's Disease Research Center, University of Wisconsin School of Medicine and Public Health, University of Wisconsin-Madison, Madison, WI, USA

**Keywords:** Alzheimer's disease, biomarkers, cerebrospinal fluid, idiopathic normal pressure hydrocephalus, neurodegeneration, tau proteins

## Abstract

**Background:**

Ventricular cerebrospinal fluid (CSF) was analysed peri- and postoperatively to elucidate the pathophysiology of Idiopathic normal pressure hydrocephalus (iNPH).

**Objective:**

To capture the dynamics of biomarkers and their relation to clinical symptoms.

**Methods:**

In 113 consecutively diagnosed patients, the Hellström iNPH scale was used to quantify symptom burden pre- and postoperatively. CSF was collected at shunt insertion and postoperatively by shunt reservoir puncture, and analyzed for concentrations of GFAP, YKL40, MCP-1, NfL, Aβ_40_, sAβPPα, sAβPPβ, GAP43, Alzheimer's disease biomarkers Aβ_42_, Aβ_42/40_, total tau (T-tau), phosphorylated tau (P-tau), and neurogranin.

**Results:**

Concentrations increased postoperatively for Aβ_40_ (134%), Aβ_42_ (106%), sAβPPα (112%), sAβPPβ (83%), NfL (128%), YKL40 (86%), GAP43 (124%), and MCP-1 (5%) (p < 0.001, MCP-1 (p = 0.03)), while mean concentration reductions were seen in T-tau (32%), GFAP (31%), neurogranin (49%), and Aβ_42/40_ (10%) (p < 0.001). A higher perioperative concentration of AβPPβ correlated with less pronounced gait disturbance (R_p_ 0.20 (0.01–0.38) (95% CI)), whereas higher levels of NfL (−0.23 (−0.41–(−)0.04) and MCP-1 (−0.21 (−0.37–(−)0.01)) correlated with impaired cognition. Higher MCP-1 correlated with a lower balance domain score (−0.20 (−0.37–(−)0.01)). Postoperative increases in levels of Aβ_40_ (R_s_ 0.27 (0.05–0.46)), Aβ_42_ (R_s_ 0.24 (0.02–0.44)) and YKL40 (R_s_ 0.22 (−0.00–0.43)) correlated with gait improvement, and a postoperative increase in Aβ_40_ (R_s_ 0.36 (0.05–0.60)) was associated with improvement in urinary continence (p 0.01–0.05).

**Conclusions:**

CSF biomarker concentrations change after shunt insertion. These changes, seen as increased concentrations for some biomarkers and decreased concentrations for others, are associated with improvement in core clinical symptoms and may illustrate reversibility of pathophysiological mechanisms in iNPH.

## Introduction

Idiopathic normal pressure hydrocephalus (iNPH) is a cerebrospinal fluid (CSF) disorder of mostly unknown pathophysiology with the core clinical symptoms of gait disturbance, balance impairment, urinary incontinence and cognitive decline.^1^ Shunt surgery is the current treatment option, with a reported improvement of 80 percent.^2–4^ In Sweden, the disease prevalence is suggested to be 2% of the population over 65, with a current incidence of shunt surgery as iNPH treatment of 2.2/100 000 per year.^5–7^

Mirroring cellular processes, biomarkers are of interest among neuroscientists trying to understand the disease pathophysiology further. Due to its proximity to areas of interest in iNPH research, the composition of ventricular CSF could reflect pathophysiologically relevant processes in periventricular matter.^1^ A diagnostic model has been proposed, consisting of total tau, amyloid-β40 (Aβ_40_), and monocyte chemoattractant factor 1 (MCP-1) with high specificity for iNPH.^8^ However, perioperative sampling of ventricular CSF to measure these markers has not been connected to shunt response.^9^ Aβ_40_, Aβ_42_, and amyloid-β protein precursor (AβPP)-derived proteins have been found to increase after shunting in CSF samples, possibly explained by an increase in CSF turnover by glymphatic activation.^10–13^ In the study by Jeppsson et al., total tau (T-tau) was shown to decrease in concentration, while phosphorylated tau (P-tau) and neurofilament light (NfL) increased postoperatively.^10^ The mechanisms underlying these changes remain unknown.

Alzheimer's disease (AD) and vascular disease, including subcortical small vessel disease, are common comorbidities in iNPH.^14^ The biomarker pattern for AD is comprises reduced Aβ_42_ and a low Aβ_42/40_ ratio, as well as increased T-tau, P-tau and neurogranin, compared with healthy controls.^15,16^ A hypothesis concerning differences in pathophysiology has been suggested in relation to CSF dynamics, where AD presents with CSF production failure, while iNPH presents with increased CSF outflow resistance in the periventricular matter.^17^ A randomized trial of 215 AD patients showed no advantage of low-flow shunt treatment.^18^ Comorbidity with signs of AD pathology was associated with reduced shunt response among iNPH patients according to some studies, whereas we reported no effect of biomarker evidence of AD pathology on clinical improvement.^9,19–21^ McGovern et al. explored the relationship between CSF biomarkers of amyloid and tau pathology in addition to cortical biopsies of amyloid plaques and tau tangles, showing equal cognitive function postoperatively regardless of biopsy and biomarker pattern.^22^ The sole predictive factor in their study of 52 patients was the Rey Auditory Verbal Learning Test-L (RAVLT) cognitive assessment. Lukkarinen et al. investigated Aβ+ and Aβ− patients with iNPH with regard to their biomarker pattern (Aβ_42_, T-tau, P-tau, neurogranin, and NfL) before and after surgery at 0, 3, 6 and 18 months follow-up.^11^ Aβ_42_ decreased more prominently among Aβ+ patients when analyzed in lumbar CSF. Total tau, P-tau and neurogranin increased after shunting, while NfL showed a surge, though later declining in concentration. The reason for the increase in total tau, P-tau and neurogranin was postulated to be due to outflow after shunting but the precise mechanism remains unclear. The increase was not associated with cognitive decline.

In a previous study, focusing on perioperative biomarker concentrations in ventricular CSF, we showed that the postsynaptic biomarker neurogranin was associated with improvement in the total iNPH scale score, theoretically due to synaptic plasticity.^9^ A bimodal distribution of Aβ_42/40_ ratio was noted, probably reflecting a positive or negative Aβ pathology status, without impact on shunt response.

In this study, we aimed to explore changes in ventricular CSF biomarkers reflecting a range of pathological mechanisms to mirror events of biomarker dynamics peri- and postoperatively in regard to neuronal degeneration (NfL), axonal white matter damage (T-tau), AD tau pathology (P-tau), astrogliosis (MCP-1, glial fibrillary acidic protein (GFAP), and chitinase-3-like-protein (YKL40)), and proteins of the amyloid cascade (sAβPPα, sAβPPβ, Aβ_40_, Aβ_42_, and Aβ_42/40_ ratio), with a special focus on synaptic markers growth-associated protein 43 (GAP43) (a presynaptic biomarker) and neurogranin (a postsynaptic biomarker) and their possible associations with core clinical symptoms. Specifically, we aimed to investigate postoperative changes in ventricular CSF biomarkers induced by shunt surgery. Another aim was to investigate associations between postoperative changes in ventricular CSF biomarker concentrations and postoperative changes in core clinical symptoms of iNPH, with symptom evaluation at baseline and postoperatively. The hypothesis was that levels in biomarker concentrations change after shunt surgery and are correlated to changes in symptom burden.

## Methods

### Study design and participants

The Gothenburg POiNT study comprising patients diagnosed with iNPH according to international guidelines and subjected to shunt surgery was conducted with inclusion between 2014 and 2017 at two sites in Sweden, details of which have been described elsewhere.^9,23^ Diagnostics were conducted according to the Relkin 2005 guidelines by a team of experienced in iNPH, including neurologists, physiotherapists, neuropsychologists and nurses.^24^ Complex cases where the diagnosis was considered uncertain, mostly due to indistinct or atypical symptomatology, were subject to the lumbar CSF tap-test or a lumbar infusion test to support the diagnosis prior to decision of surgery. Clinical testing was graded according to the Hellström iNPH scale.^25^ Lumbar puncture was performed according to standard protocol, in difficult cases complemented by a CSF tap test and/or a lumbar infusion test. Of 272 patients assessed for eligibility in the POiNT study, 143 patients with a diagnosis of iNPH, who had been treated by shunt surgery and postoperatively assessed without evidence of major comorbidities influencing the postoperative assessment, were included in this retrospective cohort.^23^ For the present paper, patients were excluded if lacking perioperative (n = 24) or postoperative (n = 6) ventricular CSF samples. Perioperative and postoperative CSF samples were available for 113 patients who were thus included in this study. Shunt surgery was performed a median of 109 (IQR 70–133) days after the diagnostic work-up. Either ventriculo-peritoneal (n = 109) or ventriculo-atrial (n = 4) shunts were used. Shunt patency was examined by radionucleid shuntography and/or lumbar infusion test if patients were unimproved upon follow-up. Follow-up was conducted a median of five (IQR 4–7) months postoperatively. All shunts were deemed working at the follow-up.

### Outcomes

Symptom evaluation was conducted pre- and postoperatively by trained personnel, with grading using the validated iNPH scale introduced by Hellström, yielding a possible total score of 0–100, 100 representing the performance of healthy controls.^25^ The symptom domains gait, balance, cognitive and continence, which also scored 0–100 points, were evaluated separately in addition to the total score. Change in iNPH scale score was referred to as delta score and calculated by postoperative minus preoperative scoring. Patients were deemed improved if the total delta iNPH score was ≥5.^25^

### Procedures

Perioperative sampling of CSF was conducted immediately after shunt insertion. After the first 2 ml of CSF was discarded, 8 mL was collected. Postoperative ventricular CSF was collected during the postoperative follow-up through 23 G/0.6 mm needle puncture of the Rickham reservoir, which was placed right frontally, with the collection of 8 mL CSF. Changes of biomarker concentrations over time through Rickham puncture has been described elsewhere.^11^ All CSF samples were aliquoted and stored in a freezer at −80 until analysis. CSF was analyzed by trained and board-certified laboratory personnel blinded to clinical data at the Neurochemistry Laboratory at Sahlgrenska University Hospital. Aβ-related biomarkers (Aβ_40_, Aβ_42_, sAβPPα, and sAβPPβ) and MCP-1 were analyzed by electrochemiluminescence assays (Meso Scale Discovery, Rockville, MD, USA). Validated in-house ELISA methodology was used to analyze NfL, neurogranin, and GAP-43, whereas CSF levels of T-tau, P-tau, and GFAP were measured using commercially available Lumipulse technology (Fujirebio, Ghent, Belgium), as previously described. YKL-40 was measured using Human Chitinase 3-like 1 Quantikine ELISA Kit (R&D Systems, Minneapolis, MN).^26–29^ All concentrations are given in ng/L. One round of experiments was conducted using one batch of reagents. Internal quality control samples were monitored at the beginning and end of each run; intra-assay coefficients of variation were below 10%.

### Statistical analysis

Descriptive statistics of participant demographics are presented using the mean ± standard deviation (SD) or median and interquartile range (IQR) for numeric variables. Categorical variables are presented in numbers and percentages. A missing case analysis was conducted to capture possible selection bias.

CSF concentration data were log-transformed prior to analysis and presented using the geometric mean and coefficient of variation (CV; the ratio of the SD to the mean), as appropriate for strictly positive and right-skewed data. The geometric mean was calculated as $e^{\mu }$ , where µ is the logarithmic mean, and the CV was calculated according to a log-normal model as $100\sqrt{e^{\;\sigma^{2}}-1}$ , where σ is the logarithmic SD. An exception was made for the amyloid ratio (Aβ_42/40_), which showed a bimodal rather than log-normal distribution and hence was analyzed on the original scale and presented as mean ± SD for the whole group.

Perioperative-postoperative changes in biomarkers were evaluated using a paired T-test, after appropriate transformations (i.e., log-transformation of all biomarkers except Aβ_42/40_). Subdomain scores were also transformed logarithmically. The results are presented as fold-changes (log-transformed data) or mean changes (non-transformed data) with 95% confidence intervals. The fold change was calculated as $e^{\delta }$ , where δ is the logarithmic mean difference. Bivariate correlation analyses between biomarkers were performed using Pearson correlation coefficients (*r*), after appropriate transformations. Correlations with subdomain scores were evaluated using Spearman rank correlation coefficients (*r_S_*). Adjustments were made for age, delay to surgery, sex, and vascular risk factors (prevalence of heart disease, hypertension, or diabetes mellitus) using corresponding partial correlation coefficients.

The dynamics of all biomarkers (delta) were included in a multivariable regression model with change in total iNPH scale score as the primary outcome variable. A ratio of P-tau/Aβ_42_, as described by Vanninen et al., was constructed to mirror AD pathology.^30^ All statistical tests were two-sided and conducted at the 5% significance level. Statistical analyses were performed by using IBM SPSS Statistics, version 29.0 (IBM Corp, Armonk, NY, USA).

## Results

The median age was 74 years and 66% were male. In all, 72% of the patients were improved a median of five (IQR 4–7) months after surgery, and improvements were seen in all the subdomains: gait, balance, cognition and continence (p = <0.001–0.064) (Table 1). A missing case analysis showed that included patients had higher preoperative and postoperative scores on the total iNPH scale score compared to those excluded. The preoperative scores for included patients had a mean of 55 ± 17 (mean difference 7, 95% CI 0–14, p = 0.046, independent samples T-test). The postoperative scores for included patients showed a mean of 57 ± 23 (mean difference 10, 95% CI 2–17, p = 0.010, independent samples T-test), while the mean for excluded patients was 67 ± 17.

**Table 1.** Demographic and clinical data of 113 iNPH patients.

	All patients	Improved	Unimproved	P
	n = 113	n = 81 (72%)	n = 32 (28%)	
Duration of symptoms (months)	36 (21–48)	36 (23–49)	30 (10–41)	1.00*
Time from diagnosis to surgery (days)	109 ± 55	107 ± 52	115 ± 66	0.47^§^
Age (y)	74 ± 7	74 ± 6	76 ± 8	0.26^§^
Sex (men)	n = 76 (67%)	n = 52 (%)	n = 24 (%)	0.37^‡^
Vascular risk factors: heart disease, hypertension, or diabetes mellitus (yes)	n = 84 (71%)	n = 28 (74%)	n = 56 (69%)	0.671^‡^
Preoperative Gait score (mean ± SD)	48 ± 25	46 ± 24	51 ± 27	0.106*
Postoperative Gait score (mean ± SD)	64 ± 26	69 ± 24	52 ± 27	0.012*
Delta Gait (mean ± SD)	16 ± 17	23 ± 15	1 ± 8	<0.001*
Preoperative Cognitive score (mean ± SD)	49 ± 21	50 ± 20	47 ± 22	0.55^§^
Postoperative Cognitive score (mean ± SD)	57 ± 21	59 ± 21	51 ± 20	0.064^§^
Delta Cognitive score (mean ± SD)	8 ± 10	9 ± 9	4 ± 10	0.007§
Preoperative Balance score (mean ± SD)	61 ± 21	61 ± 22	63 ± 19	0.71*
Postoperative Balance score (mean ± SD)	71 ± 14	72 ± 15	65 ± 17	0.006*
Delta Balance score (mean ± SD)	9 ± 16	12 ± 16	2 ± 11	0.002*
Preoperative Continence score (mean ± SD)	60 ± 26	60 ± 25	62 ± 28	0.21*
Postoperative Continence score (mean ± SD)	74 ± 26	80 ± 23	63 ± 28	0.067*
Delta Continence score (mean ± SD)	14 ± 23	20 ± 23	0 ± 11	<0.001*
Preoperative iNPH scale score	55 ± 17	53 ± 17	58 ± 18	0·16§
Postoperative iNPH scale score	67 ± 17	70 ± 16	50 ± 3	0·002§
Delta iNPH scale score	13 ± 11	17 ± 4	1 ± 4	<0·001§

Mean (± SD) or median (IQR) is given. MMSE: Mini-Mental State Examination. Unimproved patients defined by a delta iNPH scale score of <5, while a score of ≥5 indicated improvement. p-values represent comparisons across groups of unimproved and improved patients. *Mann Whitney U test; ^§^Student's T-test; ^‡^Chi^2^ test.

Biomarker concentrations are shown in Table 2.

**Table 2.** Biomarker levels in peri- and postoperative ventricular CSF of iNPH patients and change in concentration in regard to perioperative concentration values.

	Perioperative	Postoperative		
	Geometric mean (CV, %)		Fold change (95% CI)	p
Aβ_40_ (ng/L)	3726 (58%)	8730 (47%)	2.34 (2.15, 2.54)	<0.001
Aβ_42_ (ng/L)	297 (62%)	623 (69%)	2.06 (1.88, 2.26)	<0.001
AβPPα (ng/L)	73 (78%)	156 (64%)	2.12 (1.91, 2.34)	<0.001
AβPPβ (ng/L)	197 (61%)	365 (53%)	1.83 (1.69, 1.99)	<0.001
P-tau (ng/L)	51 (68%)	54 (63%)	1.04 (0.89, 1.21)	0.61
T-tau (ng/L)	610 (89%)	410 (63%)	0.68 (0.57, 0.81)	<0.001
NfL (ng/L)	925 (63%)	2001 (66%)	2.28 (2.05, 2.53)	<0.001
GFAP (ng/L)	996 (77%)	681 (47%)	0.69 (0.60, 0.79)	<0.001
YKL40 (ng/L)	97 (48%)	182 (44%)	1.86 (1.75, 1.98)	<0.001
MCP-1 (ng/L)	454 (34%)	469 (42%)	1.05 (1.02, 1.08)	0.003
Neurogranin (ng/L)	362 (67%)	183 (46%)	0.51 (0.44, 0.59)	<0.001
GAP43 (ng/L)	2254 (60%)	4933 (63%)	2.24 (1.95, 2.57)	<0.001
	Mean (SD)		Mean difference (95% CI)	
Aβ_42/40_	0.082 (± 0.018)	0.076 (± 0.026)	−0.007 (−0.009, −0.004)	<0.001

Descriptive data are presented using the geometric mean and coefficient of variation (%) or mean and standard deviation.

All biomarkers except amyloid ratio (Aβ_42/40_) were log-transformed prior to analysis. Perioperative-postoperative changes were evaluated using the paired T-test after appropriate transformations. The results are presented as the fold change (log-transformed variables) or mean change (amyloid ratio) from perioperative to postoperative ventricular CSF.

The biomarker concentrations were increased for Aβ_40_, Aβ_42_ AβPPα, AβPPβ, NfL, YKL40, MCP-1, and GAP43 postoperatively compared to perioperatively. There was a 2.34-fold increase (95% CI 2.15–2.52 / 134% increase, 95% CI 115–152%) in Aβ_40_ from periop to post-op (p < 0.0001). There was an 83% increase in AβPPβ (95% CI 69–99%) from periop to post-op (p < 0.0001). The biomarker concentrations were reduced for Aβ_42/40_ ratio, T-tau, GFAP, and neurogranin. There was a 31% reduction (95% CI 21–40%, p < 0.001) in GFAP. The phosphorylated tau concentration did not change after surgery.

The correlation of postoperative change in CSF biomarker concentrations to postoperative improvement by total iNPH scale score is presented in Table 3, with adjustments for potential patient characteristic bias of age, sex, delay to surgery and prevalence of cardiovascular risk factors. Adjustment of regression to the mean did not contribute to outcome prediction.

**Table 3.** Correlation analyses of delta iNPH total scale score related to peri-postoperative changes in biomarker concentrations, unadjusted and adjusted for age, sex, time to surgery and cardiovascular risk factors.

	Pearson correlation coefficient (*r*) (95% CI)			
	Unadjusted	*p*	Adjusted	*p*
Aβ_40_	0.15 (−0.05, 0.33)	0.15	0.13 (−0.11, 0.36)	0.20
Aβ_42_	0.13 (−0.07, 0.32)	0.20	0.10 (−0.12, 0.30)	0.32
Aβ_42/40_	−0.04 (−0.23, 0.16)	0.70	−0.08 (−0.27, 0.12)	0.44
AβPPα	0.11 (−0.09, 0.308)	0.29	0.09 (−0.12, 0.30)	0.36
AβPPβ	0.06 (−0.14, 0.25)	0.59	0.06 (−0.15, 0.25)	0.59
P-tau	−0.08 (−0.27, 0.12)	0.42	−0.09 (−0.35, 0.16)	0.39
T-tau	−0.10 (−0.29, 0.10)	0.35	−0.10 (−0.33, 0.11)	0.34
NfL	0.01 (−0.19, 0.20)	0.96	−0.01 (−0.23, 0.20)	0.89
GFAP	0.05 (−0.15, 0.25)	0.61	0.063 (−0.167–0.268)	0.55
YKL40	0.16 (−0.04, 0.35)	0.11	0.171 (−0.076–0.403)	0.10
MCP-1	0.11 (−0.09, 0.30)	0.28	0.098 (−0.166–0.327)	0.35
Neurogranin	−0.10 (−0.29, 0.10)	0.35	−0.121 (−0.317–0.080)	0.24
GAP43	0.07 (−0.13, 0.27)	0.50	0.056 (−0.138–0.237)	0.59

Statistical analyses were performed using Pearson correlation coefficients.

All biomarkers except amyloid ratio (Aβ_42/40_) were log-transformed prior to analysis.

In a multiple comparison analysis, whether stepwise or backward, none of the biomarkers correlated to the total iNPH scale score, with a 7.5% explanation of variance in outcome prediction.

### Associations between biomarker levels and core iNPH symptoms

The perioperative biomarker concentrations and their correlations to preoperative clinical scoring are depicted in Table 4. The correlations of dynamics in biomarker concentrations by improvement as assessed within subdomain iNPH scale scores are presented in Table 5. The postoperative biomarker concentrations in correlation with postoperative clinical scoring are depicted in Table 6.

**Table 4.** Correlation analyses of perioperative biomarker concentrations in relation to clinical domain scores preoperatively.^a^

	Spearman correlation coefficient (*r*) (95% CI)							
	Gait	*p*	Balance	*p*	Cognition	*p*	Continence	*p*
Aβ_40_	0.11 (−0.08–0.29)	0.25	0.09 (−0.10–0.30)	0.32	0.10 (−0.09–0.28)	0.30	0.05 (−0.13–0.24)	0.57
Aβ_42_	0.10 (−0.88–0.28)	0.27	0.05 (−0.16–0.22)	0.60	0.12 (−0.10–0.32)	0.21	0.06 (−0.12–0.23)	0.51
Aβ_42/40_^b^	−0.03 (−0.23–0.19)	0.77	−0.04 (−0.24–0.14)	0.64	0.04 (−0.14–0.24)	0.66	−0.01 (−0.21–0.17)	0.88
AβPPα	0.15 (−0.04–0.34)	0.10	0.11 (−0.09–0.31)	0.24	−0.01 (−0.20–0.18)	0.89	0.05 (−0.12–0.22)	0.60
AβPPβ	0.20 (0.01–0.38)	0.030	0.14 (−0.05–0.33)	0.13	0.05 (−0.13–0.23)	0.58	0.30 (−0.17–0.21)	0.75
P-tau	0.01 (−0.20–0.20)	0.94	0.07 (−0.14–0.28)	0.48	0.03 (−0.15–0.21)	0.77	−0.06 (−0.25–0.13)	0.54
T-tau	0.01 (−0.18–0.22)	0.92	0.09 (−0.10–0.28)	0.36	0.06 (−0.13–0.25)	0.52	−0.09 (−0.26–0.10)	0.36
NfL	−0.14 (−0.32–0.05)	0.13	−0.13 (−0.30–0.04)	0.18	−0.23 (−0.41–(−)0.04)	0.014	0.12 (−0.05–0.31)	0.20
GFAP	0.00 (−0.20–0.20)	0.99	0.02 (−0.19–0.22)	0.85	−0.07 (−0.26–0.12)	0.46	0.17 (−0.01–0.35)	0.073
YKL40	0.02 (−0.18–0.21)	0.87	−0.08 (−0.28–0.13)	0.41	−0.13 (−0.31–0.06)	0.19	0.10 (−0.09–0.30)	0.29
MCP-1	−0.08 (−0.28–0.11)	0.42	−0.20 (−0.37–(−0.01)	0.036	−0.21 (−0.37–(−)0.01)	0.029	0.15 (−0.04–0.34)	0.11
Neurogranin	0.01 (−0.18–0.19)	0.90	0.11 (−0.10–0.29)	0.26	0.10 (−0.09–0.29)	0.32	−0.05 (−0.23–0.14)	0.58
GAP43	0.01 (−0.19–0.22)	0.91	0.01 (−0.12–0.21)	0.93	−0.04 (−0.22–0.16)	0.70	−0.01 (−0.20–0.17)	0.95

^a^ Ventricular CSF biomarkers as independent variables, transformed by log, and log-transformed delta iNPH scale score (by domain scores) after shunt surgery as a dependent variable.

^b^ Not transformed.

**Table 5.** Correlation analyses between change in ventricular biomarkers (postoperative – perioperative concentrations) in relation to change in iNPH scale score, as assessed by the four symptom subdomains of gait, balance, cognition and continence.^a^

	Spearman correlation coefficient (*r*) (95% CI)							
	Gait	*p*	Balance	*p*	Cognition	*p*	Continence	*p*
Aβ_40_	0.27 (0.05–0.46)	0.016	−0.12 (−0.43–0.22)	0.48	0.04 (−0.21–0.28)	0.78	0.36 (0.05–0.60)	0.020
Aβ_42_	0.24 (0.02–0.44)	0.030	−0.25 (−0.54–0.09)	0.14	0.01 (−0.24–0.25)	0.94	0.24 (−0.08–0.51)	0.13
Aβ_42/40_^b^	0.05 (−0.16–0.27)	0.66	−0.12 (−0.43–0.23)	0.49	−0.05 (−0.29–0.20)	0.70	−0.06 (−0.37–0.25)	0.69
AβPPα	0.16 (−0.07–0.37)	0.16	−0.16 (−0.47–0.18)	0.35	−0.04 (−0.28–0.21)	0.74	0.22 (−0.10–0.50)	0.16
AβPPβ	0.14 (−0.89–0.35)	0.22	−0.02 (−0.35–0.32)	0.91	0.09 (−0.16–0.33)	0.46	0.13 (−0.19–0.42)	0.42
P-tau	−0.01 (−0.23–0.22)	0.95	0.22 (−0.12–0.51)	0.20	0.05 (−0.20–0.29)	0.67	0.10 (−0.22–0.40)	0.53
T-tau	−0.03 (−0.25–0.19)	0.78	0.27 (−0.07–0.55)	0.11	0.06 (−0.19–0.30)	0.62	0.17 (−0.15–0.46)	0.29
NfL	−0.02 (−0.24–0.21)	0.89	−0.11 (−0.43–0.23)	0.51	0.05 (−0.20–0.29)	0.68	0.13 (−0.19–0.43)	0.40
GFAP	0.11 (−0.12–0.32)	0.35	−0.17 (−0.48–0.18)	0.33	−0.02 (−0.26–0.23)	0.88	0.18 (−0.15–0.47)	0.26
YKL40	0.22 (0.00–0.43)	0.046	−0.23 (−0.52–0.12)	0.18	0.12 (−0.13–0.36)	0.32	0.19 (−0.13–0.48)	0.23
MCP-1	−0.06 (−0.28–0.17)	0.61	−0.06 (−0.38–0.28)	0.73	0.16 (−0.09–0.39)	0.21	−0.05 (−0.36–0.27)	0.78
Neurogranin	−0.06 (−0.28–0.17)	0.62	0.22 (−0.12–0.51)	0.20	0.03 (−0.21–0.28)	0.78	0.10 (−0.22–0.40)	0.53
GAP43	0.16 (−0.07–0.38)	0.15	−0.04 (−0.37–0.30)	0.82	0.07 (−0.18–0.31)	0.59	0.17 (−0.16–0.46)	0.30

^a^ Ventricular CSF biomarkers as independent variables, transformed by log, and log-transformed delta iNPH scale score (by domain scores) after shunt surgery as a dependent variable. ^‡^By Spearman r_s_.

^b^ Not transformed.

**Table 6.** Correlation analyses of postoperative biomarker concentrations in relation to clinical domain scores postoperatively.^a^

	Spearman correlation coefficient (*r*) (95% CI)							
	Gait	*p*	Balance	*p*	Cognition	*p*	Continence	*p*
Aβ_40_	0.11 (−0.09–0.31)	0.24	0.61 (−0.15–0.24)	0.61	0.13 (−0.06–0.32)	0.17	0.14 (−0.04–0.33)	0.16
Aβ_42_	0.07 (−0.12–0.27)	0.45	0.04 (−0.14–0.24)	0.68	0.15 (−0.04–0.33)	0.12	0.07 (−0.13–0.26)	0.47
Aβ_42/40_^b^	0.03 (−0.17–0.22)	0.72	0.03 (−0.17–0.20)	0.79	0.08 (−0.11–0.27)	0.39	0.01 (−0.17–0.19)	0.91
AβPPα	0.13 (−0.05–0.32)	0.16	0.12 (−0.08–0.32)	0.23	0.08 (−0.10–0.26)	0.41	0.07 (−0.11–0.27)	0.45
AβPPβ	0.21 (0.02–0.38)	0.023	0.18 (−0.04–0.36)	0.066	0.15 (−0.03–0.32)	0.13	0.11 (−0.08–0.28)	0.28
P-tau	0.02 (−0.18–0.21)	0.87	−0.03 (−0.22–0.17)	0.77	−0.09 (−0.28–0.10)	0.36	0.15 (−0.05–0.35)	0.11
T-tau	0.03 (−0.17–0.23)	0.75	−0.02 (−0.21–0.17)	0.84	−0.11 (−0.29–0.06)	0.26	0.11 (−0.08–0.30)	0.25
NfL	−0.23 (−0.42–(−)0.03)	0.014	−0.18 (−0.35–0.00)	0.058	−0.24 (−0.43–(−)0.05)	0.010	0.08 (−0.13–0.28)	0.44
GFAP	−0.14 (−0.31–0.04)	0.13	−0.13 (−0.30–0.08)	0.19	−0.20 (−0.36–(−)0.04)	0.035	0.08 (−0.11–028)	0.42
YKL40	−0.03 (−0.22–0.16)	0.78	−0.08 (−0.28–0.10)	0.40	−0.16 (−0.34–0.03)	0.097	0.09 (−0.12–0.29)	0.36
MCP-1	−0.11 (−0.31–0.09)	0.25	−0.20 (−0.36–0.00)	0.042	−0.26 (−0.42–(−)0.07)	0.007	0.11 (−0.07–0.31)	0.26
Neurogranin	0.08 (−0.12–0.27)	0.41	−0.04 (−0.23–0.15)	0.70	0.02 (−0.17–0.23)	0.87	0.13 (−0.07–0.31)	0.19
GAP43	0.05 (−0.15–0.25)	0.64	0.00 (−0.19–0.20)	0.97	−0.04 (−0.23–0.17)	0.72	0.16 (−0.04–0.38)	0.11

^a^ Ventricular CSF biomarkers as independent variables, transformed by log, and log-transformed postop iNPH scale score (by domain scores) after shunt surgery as a dependent variable. ^‡^By Spearman r_s_.

^b^ Not transformed.

The perioperative values of NfL correlated with preoperative cognitive assessment but did not correlate with cognitive improvement after surgery (p = 0.51) or improvement in RAVLT score (p = 0.20).

The perioperative levels of synaptic proteins did not contribute to cognitive outcome: GAP43 (p = 0.52) and neurogranin (p = 0.51).

### Amyloid pathology

The postoperative levels of the amyloid ratio (Aβ_42/40_) were not correlated to improvement in total iNPH scale score (r = 0.09, 95% CI −0.10 to 0.27, p = 0.35). Increasing age was associated with decreasing amyloid ratio perioperatively (r_p_ −0.27 (95% CI −0.43–(−)0.09) p = 0.003) and postoperatively (−0.32 (−0.48–(−)0.14) p < 0.001). The decreasing amyloid ratio after shunt surgery also correlated with increasing age (−0.25 (−0.42–(−)0.05) p = 0.013)

The perioperative levels of the amyloid ratio correlated with the postoperative RAVLT score, a cognitive test included in the iNPH scale (r_s_ _=_ 0.224 (0.025–0.399) p = 0.019 by Spearman correlation) although not with the cognitive subdomain of the iNPH scale (r_s_ p = 0.26 by Spearman correlation). A ratio of P-tau/Aβ_42_ was not correlated with shunt response, either perioperatively, postoperatively or in regard to change in ratio after shunt therapy (Pearson's p = 0.194–0.672).

## Discussion

This study explores the dynamics of CSF biomarkers induced by shunt surgery, and contributes to the understanding of iNPH pathophysiology, suggesting a novel view of biomarker proteins in regard to improvement in core symptoms. The biomarker levels were dynamically changed after shunting for all analyzed biomarkers except P-tau. These opposite changes of different biomarker levels postoperatively suggest that no general wash-out effect induced by the shunt system seems to occur. The postoperative concentrations decreased for the Aβ_42/40_ ratio, T-tau, neuroinflammatory protein GFAP and postsynaptic protein neurogranin, while other biomarkers increased in concentration. Restoration of amyloid metabolism appears to relate to gait and urinary continence improvement, captured by an Aβ_40_ and Aβ_42_ increase for patients with a more pronounced gait subdomain score increase and an Aβ_40_ increase among patients with improved continence. Neural injury, indicated by NfL levels, may contribute to impairment in cognition and gait, as observed in the postoperative gait subdomain evaluation, and cognitive evaluation conducted peri- and postoperatively. Neuroinflammation may play a role in balance and cognitive dysfunction and was also noted in this study to be correlated with gait improvement somehow. In this material, contrary to some symptom domain subscores, we found no single biomarker associated with improvement in the total iNPH scale score, perhaps due to diverse smaller alterations in brain chemistry connected to certain symptom dynamics. Additional biomarkers could be included in future studies to explore if there are specific biomarker patterns associated with overall shunt response.

In line with previous data, postoperative concentrations NfL were increased compared to perioperative levels while T-tau decreased, where the decrease of tau may indicate that cellular damage has not yet occurred, a rapid restitution of tau prior to follow-up sampling, or it may be a sign of increased metabolism after shunt treatment.^10^ The increase in NfL may reflect the effect of neurological surgery, although the minute trauma to the parenchyma, with NfL as a neuronal damage marker. Aβ_40_ presented with the steepest postoperative increase of 134%, while Aβ_42_ increased by 106%. The steeper increase in Aβ_40_ compared to Aβ_42_ somewhat contradicts earlier findings (showing an increase of Aβ_40_ 116% and Aβ_42_ 144%^10^). In comparison to preoperative levels in lumbar CSF, Aβ_42_ has been found to increase as well as remain unaltered in iNPH.^31,32^ We suggest the higher increase here of Aβ_40_ explains the total reduction in the Aβ_42/40_ ratio, but otherwise this is contradictory to improvement as a low ratio is suggested to mirror amyloid plaque burden.

The prediction of shunt response has been investigated previously, with varying results. Some have found that biomarkers do not contribute to the prediction of outcome.^13^ Here, changes in biomarker concentrations did not contribute to the overall response to shunt treatment. A combination of all biomarkers in a multivariate regression did not contribute further to outcome prediction for the total symptom burden relief.

Reduced levels of precursor proteins of the amyloid cascade (sAβPP) and Aβ were seen in iNPH patients compared to healthy controls, which was interpreted as a sign of reduced amyloid metabolism due to sAβPP production decrease or in combination with the reduced clearance of molecules by iNPH-specific retrograde CSF dynamics.^8,33^ Some studies suggest sAβPP are decreased in shunt responders, while others suggest an increase.^10^ Amyloid proteins in ventricular CSF have previously been found to increase after shunt treatment, which was also shown here. Low levels of Aβ_42_ have previously been described as an indicator of poor shunt response due to increased amyloid plaque burden, and as a biomarker indicator of plaques the Aβ_42_ is inversely correlated with increasing plaque burden.^34,35^ The relative increase in iNPH patients could still be considered low when compared to AD or healthy controls.^8^ Ultimately, the suggested restoration of amyloid metabolism here seems to correlate with improvement, with high levels in precursor protein AβPPβ as well as increases of Aβ_40_ and Aβ_42_ to explain the reduced symptom burden, in line with the findings described by Jeppsson et al. (2013, 2016) where amyloid proteins were decreased in ventricular CSF perioperatively and increased postoperatively, although the findings were not considered in the light of possible subdomain symptom burden there.^10,36^ Some suggest low Aβ_40_ is connected to gait.^37^ Here, AβPPβ levels were increased levels among patients with better gait upon diagnosis and at follow-up, and increasing improvement in gait and urinary continence was shown with a greater increase in Aβ_40_ and Aβ_42_. AβPPβ, a biomarker also suggested to have a role in synaptic plasticity, could also suggest a synaptic mechanism for gait impairment, although synaptic biomarkers GAP43 and neurogranin did not contribute to improvement, which challenges the theory.^10^

NfL, a biomarker mirroring axonal injury in subcortical areas in periventricular matter, was elevated after surgery here (128%).^10,12,13,32^ A greater decrease in NfL, with minimized axonal injury has been correlated with improvement of gait and balance.^37^ When comparing iNPH to healthy controls, NfL has been reported to be elevated as well as decreased.^21^ As explained by Lukkarinen et al., the surge in NfL was seen in the first month after surgery, later declining in concentration, possibly illuminating how an increase is still shown after 5.4 months have passed since shunt insertion.^11^ Increased levels of NfL, captured perioperatively here, correlated inversely with cognitive scoring, suggesting increased axonal injury burden subcortically in patients with more prominent cognitive impairment. However, the surgical procedure could possibly also result in a surge of T-tau as is the case in other brain injuries, not seen here. This might be due to a delayed surge, as presented by Lukkarinen et al. in lumbar CSF, however not in ventricular CSF or a more rapid restitution to baseline levels for T-tau than for Nfl.^11^ An elaboration of the study with increased length of follow-up, could elucidate postoperative dynamics further.

Increased MCP-1 at the point of surgery correlated inversely with balance and cognitive scoring, suggesting glial activation and neuroinflammation to be part of the pathophysiology behind balance and cognitive impairment. Another marker of astrogliosis, YKL40, may also contribute to gait improvement as concentrations increased in parallel to shunt response. Previous data did not show dynamics in the neuroinflammation captured by MCP-1, while we noted an increase in MCP-1 of 5%, and YKL40 of 86%, while GFAP was reduced by 31%.^10^ MCP-1 has been found to increase in iNPH as a sign of neuroinflammation and neuroglia activation when compared to patients with other dementia disorders (MCI, AD, frontotemporal dementia) and healthy controls.^38^ Others suggest the MCP-1 increase is related to unfavorable shunt response, with signs of non-beneficial inflammation perhaps due to the shunt itself.^39,40^ Decreased astrogliosis (reduced levels of GFAP) has also been described to correlate with favorable outcomes.^21^ The shunt insertion itself may induce transient inflammation, resulting in the surge of two inflammatory biomarkers, possibly also explaining the connection of YKL40 increase to gait improvement. Overall, higher concentrations of inflammatory biomarkers MCP-1 and GFAP explained more cognitive impairment and lower balance scores both pre- and postoperatively. The mechanisms of neuroinflammation, found here to associate with symptom burden in iNPH, should be further investigated to elucidate the role of astrogliosis, glial activation and inflammation in iNPH pathophysiology.

Some studies suggest P-tau to be unchanged or lower in iNPH patients compared with healthy controls.^12,32,41,42^ Regarding P- and T-tau, concentrations were higher for non-responders to shunt treatment in a meta-analysis by Thavarajasingam et al.^43^ The prevalence of comorbid AD may influence improvement inversely.^44–46^ Vanninen et al. described a ratio of P-tau/Aβ_42_ of 0.013 with an AUC of 79% when diagnosing comorbid AD, but we found no association between shunt response and the P-tau/Aβ_42_ ratio.^30^ Gait may be influenced by lower concentrations of P- and T-tau although this association was not found in our study.^37^ Amyloid plaque formation, seen as a low ratio of Aβ_42/40_, was not associated with improvement, further stressing that improvement can occur regardless of signs of amyloid pathology. Improvement in RAVLT, a test for memory function, was weakly associated with increased preoperative vCSF Aβ_42/40_ ratio, perhaps due to signs of less amyloid plaque burden. T-tau and P-tau, described previously to predict poor shunt response when increased, did not contribute to outcome prediction in our study.^34^

The association between age and amyloid ratio reduction, where increasing age was associated with greater reduction of the ratio, could suggest that the aging brain is at increased risk of amyloid plaque formation, maybe due to defective clearance mechanisms.^47^ Aβ_42_ and P-tau concentrations correlate with age in iNPH patients, further adding to this hypothesis.^48^

An increase in biomarker concentration could mirror increased biomarker production, or glymphatic activation with an increase in clearance of waste products and proteins to the CSF compartment while the contrary, a reduction of biomarkers, could possibly be the result of decreased production or impaired glymphatic function. The outflow contributed by the inserted shunt decreases the strain on the periventricular matter, allowing clearance of extracellular fluid and proteins, resulting in a possible wash-out effect postoperatively. To further elucidate pathophysiological processes within the brain parenchyma and how these relate to changes induced by shunt surgery in iNPH, more studies need to be conducted capturing differences in extracellular space and ventricular CSF.

As this is considered an exploratory study, no corrections for multiple comparisons have been conducted to minimize the risk of type II error. Based on a sample of 113 patients, we believe our findings could be considered robust. The correlations, although significant, should be considered with caution, as they are quite weak, which could be considered a limitation of this study. A missing case analysis demonstrated that patients included in the study had higher total iNPH scale scores pre- and postoperatively compared to those excluded, suggesting that iNPH patients with more severe symptoms may have been underrepresented, which may affect the effect size of the findings. This potential selection bias should be taken into consideration when interpreting our findings. In addition, CSF could possibly have been collected during different time points of the day, which may induce circadian differences and subsequently slightly affect our results.^49^ Further studies are suggested to improve understanding of both the events within the parenchyma and cellular mechanisms in iNPH.

### Conclusions

Ventricular CSF biomarker concentrations change after shunt insertion. These changes, manifested as increased concentrations for some biomarkers and decreased concentrations for others, are associated with improvement in core clinical symptoms and may illustrate the reversibility of pathophysiological mechanisms in iNPH.
